# Digitalized acoustic monitoring of lung congestion

**DOI:** 10.1186/cc10690

**Published:** 2012-03-20

**Authors:** S Lev, L Wolloch, I Kagan, M Grienv, P Singer

**Affiliations:** 1 Rabin Medical Center, Petah Tikva, Israel; 2 Deep Breeze Ltd, Or-Akiva, Israel

## Introduction

Changes in lung water are known to change breath sound acoustics [1]. Using two pig models, we observed that continuous elevation of lung sound amplitude may indicate an increase in total lung water content [2]. Here we report three cases of ventilated patients in whom continuous acoustic monitoring was done during extravascular lung water (EVLW) measurements.

## Methods

We retrospectively analyzed cases in which EVLWi (PiCCO) and other clinical parameters were measured, during continuous acoustic monitoring (VRI), using eight small sensors adhered to the anterior chest. A transmission factor (TF) was calculated, using the sound transfer function between different sensors. The TF changes in correspondence to changes in tissue density [1]. The difference in TF was calculated between recordings when pulmonary edema was observed (>7 ml/kg threshold accompanied with an increase of 2 ml/kg in the EVLWi) and when absent. Statistical analysis was made using a *t *test.

## Results

**Figure 1:**
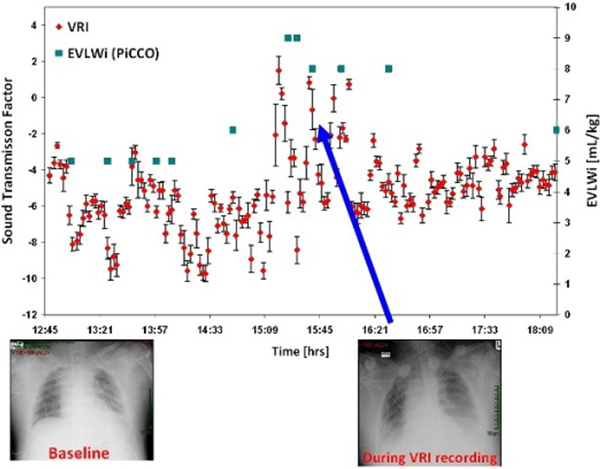
**VRI (average ± SE) versus PiCCO and CXR**.

A total of 336 continuous acoustic recordings in three patients (acoustic monitoring was applied together with EVLWi measurements) were analyzed (146 recordings when lung edema was present; 190 with no edema). In all patients, the acoustic profile corresponded to changes in the clinical picture. In two of the cases, changes in acoustic profile were similar to the ones in the EVLWi and other clinical parameters (Figure 1). In one case, where there was stability in lung sound acoustics, EVLWi and other clinical parameters were also stable. Significant differences existed between recordings with edema (-3.61 ± 0.39) and without edema (-5.71 ± 0.15) (*P *< 0.001).

## Conclusion

Changes in lung water tend to result in changes in the sound TF, due to changes in the tissue's density. These preliminary results indicate that monitoring lung sounds has the potential to monitor changes in lung water.

## References

[B1] Donnerberg Br J Dis Chest 1980 74 23 7356911

[B2] Lev Crit Care 2011 15 P174 10.1186/cc9594

